# Optimization of Wind Turbine Spindle Bearing Gel-like Grease Performance at Extreme Temperatures

**DOI:** 10.3390/gels12020161

**Published:** 2026-02-12

**Authors:** Zhenzhong Tian, Yihao Zhang, Han Peng, Budi Peng, Zihao Meng

**Affiliations:** 1 Department of Traffic Management Engineering, Henan Police College, Zhengzhou 450046, China; tzz@hnp.edu.cn; 2 School of Mechanical Engineering, North China University of Water Resources and Electric Power, Zhengzhou 450045, China; 18539131830@163.com (Y.Z.); pbd286500@163.com (B.P.); mzh000311@163.com (Z.M.); 3 School of Water Conservancy, North China University of Water Resources and Electric Power, Zhengzhou 450045, China

**Keywords:** extreme temperatures, spindle bearings, gel-like grease

## Abstract

With the advancement of wind power technology towards larger-capacity and higher-power turbines, their main shaft bearings face significant lubrication challenges under extreme temperatures. In this study, seven modified greases were prepared by adding 0.5 wt.% of tungsten disulfide (WS_2_), zinc sulfide (ZnS), and sulfurized isobutylene (T321). The concentration of all additives is given in weight percent (wt.%). Using a combined approach of friction and wear testing along with rheological analysis, this study systematically evaluated the tribological performance of the greases at high temperature (80 °C)—with the friction coefficient and wear scar diameter as key parameters—and their rheological properties across a wide temperature range (−20 °C to 80 °C), focusing primarily on shear stress and viscosity. All critical input parameters, including temperature, load, and shear rate, were precisely controlled and monitored using calibrated instruments. Results indicate that the WS_2_ and T321 compounding system demonstrated optimal performance, achieving a low average coefficient of friction of 0.024 and an average wear scar diameter of only 0.367 mm. At the same time, the WS_2_/T321 composite formulation exhibits excellent shear stability at high temperatures and good flow properties at low temperatures, demonstrating optimal viscosity–temperature characteristics. This study develops a promising grease formulation through multidimensional performance evaluation, offering key experimental support for designing high-performance wind turbine spindle bearing greases under high-temperature conditions.

## 1. Introduction

The continuous growth in energy consumption and the environmental issues arising from finite traditional energy sources constitute one of the central challenges facing modern industrial society. Nevertheless, conventional sources like coal, oil, and natural gas face not only finite reserves but also severe environmental pollution throughout their extraction and consumption [1,2]. Consequently, the pursuit of clean and renewable alternatives—such as solar, wind, and biomass—has gained global prominence, playing a vital role in curbing reliance on fossil fuels, mitigating environmental impact, and promoting sustainable development [3,4,5]. Among these, wind energy has emerged as one of the most rapidly expanding and technologically mature renewable sources, with wind turbines representing a key infrastructure for converting kinetic wind energy into electrical power. Their development and reliability are therefore central to the global energy transition. Among various renewable energy sources, wind energy has become one of the most technologically mature and economically competitive options, owing to its abundant global resources, scalability, and rapidly declining cost of energy production [6]. Wind originates from temperature differences across the Earth’s surface caused by uneven solar radiation, leading to air flow from high-pressure zones to low-pressure zones. Wind power generation harnesses this principle by converting wind energy into mechanical energy through wind turbines, thereby producing clean, green electricity [7,8,9,10].

Wind power technology converts the kinetic energy of wind into electrical energy through wind turbines, a process that is both environmentally friendly and highly efficient [11]. Wind turbines are typically located in remote and inaccessible areas such as plateaus, deserts, and coastal zones, where they must operate under multiple harsh conditions including aridity, high humidity, temperature fluctuations, and extreme environments [12]. Within the turbine, the spindle bearing serves as the critical sliding/rolling interface for energy conversion. The lubrication of this interface faces severe challenges under these harsh conditions, and its failure directly leads to increased friction, accelerated wear, and ultimately, catastrophic component failure. To ensure the safe and reliable operation of wind turbines, the main shaft bearings must possess high strength, high rigidity, excellent impact resistance, and a long service life [13,14]. However, over time, factors such as temperature variations, load fluctuations, and vibrations inevitably cause wear in bearings, thereby increasing the risk of wear-related failure [15]. Once bearing failure occurs, it not only necessitates the replacement of related components but may also trigger various safety incidents such as fires, severely disrupting the normal operation of wind turbines and resulting in substantial economic losses [16,17,18]. To reduce failure rates, lubrication is central to maintaining stable operation of spindle bearings. Selecting high-quality lubricants and employing appropriate lubrication methods can effectively ensure smooth functioning of spindle bearings [19].

Due to their unique operating conditions, wind turbine main shaft bearings typically employ high-performance gel-type grease as the lubricating medium [20,21,22]. This type of gel-like grease must meet stringent performance criteria: under extreme temperature conditions, it must not only maintain stable lubricating properties but also exhibit excellent extreme pressure anti-wear characteristics, oxidation resistance, and good flow characteristics [23,24,25]. If the gel-like grease exhibits insufficient high-temperature oxidation resistance, it is prone to oxidation thickening and coking; poor low-temperature pumpability can lead to inadequate lubrication; under prolonged shear and vibration, its colloidal structure may break down, causing oil separation or hardening. These factors can all result in the failure of the lubricating film, thereby aggravating bearing wear or even leading to premature damage. To ensure the reliability of gel-like grease in practical applications, this study employs a comprehensive evaluation method combining tribology and rheology [26,27]. The effectiveness of lubrication is fundamentally a systematic tribological issue, the core of which lies in controlling the interaction between contacting surfaces to reduce wear and energy consumption. Classical tribology encompasses in-depth research on the mechanisms of friction, wear, and lubrication. In recent years, tribological design has evolved from the singular optimization of lubricant formulations to include the active design and modification of the contacting surfaces themselves. For instance, in the field of tool machining, creating specific micro-textures on the surfaces of ceramic or superhard materials has been proven to effectively improve lubricant retention, reduce friction, and enhance machining quality [28]. These advancements indicate that solutions for specific extreme operating conditions (such as the high-temperature and heavy-load environment of wind turbine bearings) often require multi-dimensional and synergistic innovation. For the wind turbine spindle bearings focused on in this study, their enclosed structure precludes real-time surface re-engineering or the application of external auxiliary processes, unlike open machining systems. Therefore, focusing the core of performance optimization on the lubricating medium itself—i.e., developing a high-performance gel-like grease capable of maintaining a stable lubricating film across a wide temperature range—becomes the most direct and feasible technical pathway to enhance the reliability of this critical component. Based on this rationale, this study aims to strengthen the grease’s ability to form and resist the breakdown of films at the friction interface through systematic additive formulation. Among these, tribological performance testing focuses on evaluating experimental parameters such as the coefficient of friction and wear scar diameter of gel-type grease on steel balls. These parameters exert a decisive influence on the friction and wear behavior of wind turbine bearings operating under extreme temperatures over extended periods [29,30]. Systematic evaluation of the tribological properties of gel-like grease provides crucial assurance for the reliable operation of wind turbine main shaft bearings under extreme conditions [31].

To effectively reduce friction and wear in wind turbine bearings and extend their service life, lubrication technology is key to addressing bearing friction and wear issues. Wu et al. [32] evaluated the lubrication reliability of wind turbine main shafts under random wind conditions. Through simulation under random wind conditions, they found that random wind conditions significantly increase the probability of lubrication failure between the rollers and inner ring of the main shaft bearings. Furthermore, the probability of lubrication failure increases as the average wind speed decreases, exhibiting an S-shaped curve. This study provides valuable insights for evaluating the lubrication performance of wind turbine main shaft bearings under harsh conditions and holds significant importance for research on wind turbine bearing lubrication technology. Jiang et al. [33] combined the Reynolds equation, energy equation, and viscosity–temperature equation to investigate lubrication characteristics in the micro-clearance of fluid dynamic sliding bearings at high rotational speeds. Results indicate that as the eccentricity or length-to-diameter ratio increases, the maximum peak values of pressure, temperature, and heat dissipation rise rapidly, cavitation intensifies, and flow velocity accelerates to varying degrees. With increasing bearing rotational speed, the maximum temperature peak increases sharply. Li et al. [34] designed a novel piezoelectric microjet bearing lubrication device and investigated the effects of excitation parameters on microjet injection performance under different voltages. The results indicate that this device offers higher precision in determining the drop point, along with greater flexibility and efficiency. When the pulse voltage is 60 V, the operating frequency is 2.1 kHz, and the duty cycle is below 70%, superior microjet performance can be achieved. Jin et al. [35] developed a three-dimensional porous tilting pad bearing hybrid lubrication model. By integrating a grid iteration algorithm, they analyzed the influence of parameters such as bearing rotor speed, tilting stiffness, and nominal clearance on the viscoelastic properties and dynamic characteristics of tilting pad bearings. Simulation experiments reveal that under air lubrication, the temperature rise within the tilting pad bearing remains below 3 K. Under mixed lubrication, the adaptive pad movement of the tilting pad bearing exhibits favorable viscoelastic properties and dynamic characteristics. Le et al. [36] investigated the effects of lubricants and oil-gas mixtures on the temperature of machine tool spindle bearings. They used thermal imaging cameras and thermocouples to measure the temperature distribution and temperature rise rate of the bearings. Research findings indicate that compared to using gel-based lubricants, employing oil-gas mixtures proves more effective in dissipating heat and reducing bearing temperatures.

Regarding research on gel-like greases in bearings, Meijer et al. [37] proposed an in situ method for measuring the rheological properties of gel-like greases to investigate their service life in rolling bearings. They combined the pipe flow model with a power-law rheological model to analyze and determine the power-law fluid viscosity index and exponent during the aging of gel-like grease. Lugt et al. [38] developed a method to quantify the gel lubricant life performance of rolling bearings based on a gel lubricant life model for ball bearings lubricated with gel lubricant under axial loading. Lugt et al. [39] investigated the lubrication mechanism of lithium grease-lubricated ball bearings during the discharge phase, emphasizing the impact of oxidation on the service life of gel-like greases. Studies on the service life of gel-like grease in “air” and “nitrogen” environments indicate that oxidation significantly impacts its longevity, though the specific mechanism remains incompletely understood. Oxidation begins during the induction period following the depletion of antioxidants, a phase influenced by bearing operating conditions and oxygen concentration. As oxidation occurs, the loss of base oil leads to a decline in lubricating performance, which can be partially compensated for by oil release from the gel-like grease reservoir. This study highlights the importance of optimizing the service life of gel-like greases and provides theoretical support for equipment operating under extreme conditions. Liu et al. [40] employed the finite volume method in computational fluid dynamics to model and analyze gearboxes lubricated with gel-like grease, numerically investigating the fluid flow characteristics of gel-like lubricants. Simulation results reveal that during gear rotation, only localized effects occur at the bottom of the oil shell surrounding the gear. When the gel-like grease filling volume is low, distinct separation occurs between the rotating gear and the gel-like grease. Conversely, when the gel-like grease filling volume is high, significant interaction develops between the gear and the gel-like grease, with the gel-like grease circulating around the gear. This experiment has certain limitations. The influence of centrifugal force on high-speed rotating gears should be considered, and its lubrication regimen should be further optimized in subsequent research. Rosenkranz et al. [41] investigated the wear protection capabilities of different gel-like grease compositions at varying temperatures. They produced four distinct gel-like greases based on mineral base oils by varying the amounts of thickener and ZDDP added. Various grease-lubricated rolling bearing experiments were conducted across a wide temperature range of 0 to 120 °C. Experiments have shown that at low temperatures, all gel-type greases provide excellent wear protection without forming an additive-based friction film. In high-temperature tests, a ZDDP friction film was formed. Wu et al. [42] employed a fourth-order Runge–Kutta method to simulate the effects of load, gel-like grease rheological properties, and temperature on roller tilt and inclination as well as bearing slip. The results indicate that the roller tilt angle in the unloaded zone is significantly greater than that in the loaded zone, while the roller tilt angle in the unloaded zone is smaller than that in the loaded zone. As rotational speed increases, roller tilt and skewing, along with bearing slippage, become more pronounced [43,44,45].

The novelty of this study lies in the systematic optimization of additive formulations for lithium complex grease, through which an optimal gel-type grease suitable for high-temperature operating conditions of wind turbine spindle bearings was developed and selected [46]. By employing an integrated evaluation approach combining tribological and rheological analyses, the research not only demonstrates the superior performance of the synergistic WS_2_/T321 formulation but also establishes a mechanistic link between its macroscopic properties and microstructural characteristics [47,48]. This work offers direct experimental evidence and theoretical insights for the development of high-performance greases for wind turbine bearings.

## 2. Results and Discussion

### 2.1. Optimization Analysis of the Tribological Properties of Gel-like Lubricating Grease for Wind Turbine Main Shaft Bearings

#### 2.1.1. Friction Coefficient–Time Analysis of Different Samples Against Steel Balls

1. Friction Coefficient–Time Analysis of Gel-Like Lubricating Grease for Wind Turbine Main Shaft Bearings Containing a Single Additive

**Figure 1:**
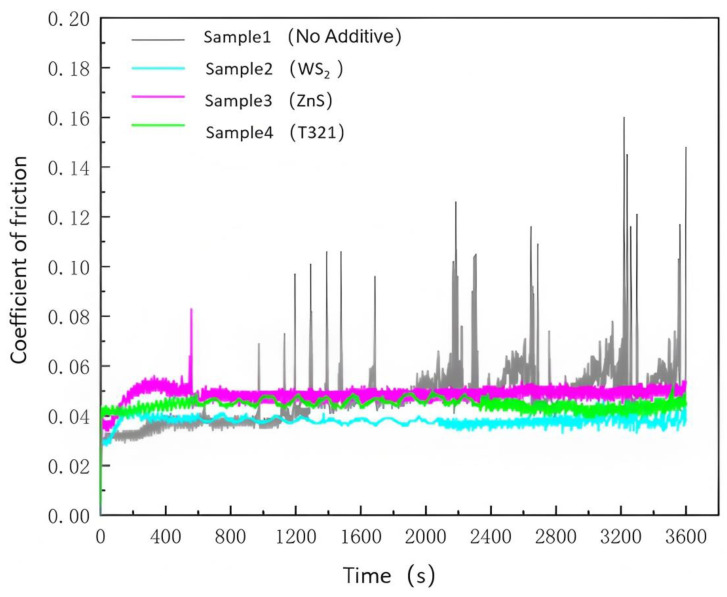
Friction coefficient–time curves of wind turbine spindle bearing grease with a single additive.

The friction coefficient–time curves of gel-type grease for wind turbine main shaft bearings containing a single additive, obtained using the MRS-10G lever-type four-ball friction and wear tester, are shown in Figure 1.

As shown in Figure 1, Sample 1 without additives maintained a coefficient of friction around 0.035 during the initial phase (0–992 s), demonstrating a certain degree of stability. However, after prolonged operation (beyond 992 s), the coefficient of friction for Sample 1 began to fluctuate frequently, reaching a maximum value of 0.16. This indicates that the lubricating performance of Sample 1 rapidly deteriorates under sustained loading. Such frequent fluctuations in the coefficient of friction not only accelerate the wear of Sample 1 but may also induce vibration or localized temperature rise, further compromising the bearing’s durability and reliability. Furthermore, this also indicates that Sample 1 exhibits poor stability, weak wear resistance, and inadequate lubrication performance after prolonged operation, thereby reducing overall reliability. These shortcomings may prevent Sample 1 from meeting the requirements for long-term stable operation in wind turbine main shaft bearings, thereby shortening its service life. Test data indicates that the average coefficient of friction for Sample 1 is 0.079.

Sample 2 maintained a low and stable coefficient of friction throughout the testing process, with a maximum value of 0.043 and an average value of 0.039. This indicates that WS_2_, as a solid lubricant, forms an effective protective film on friction surfaces, thereby reducing friction and wear. Compared to Sample 1, Sample 2 demonstrates superior lubrication performance and stability during extended operation. This stable coefficient of friction helps improve the durability of the spindle bearings and reduces energy loss caused by friction. Therefore, WS_2_ as an additive offers significant advantages in practical applications, particularly in environments requiring long-term stable operation.

Sample 3 exhibited a relatively high coefficient of friction during the initial phase (0–600 s), but subsequently stabilized. Although its anti-friction and wear resistance was not as pronounced as that of Sample 2, Sample 3 still demonstrated relative stability during prolonged testing. This indicates that ZnS can provide lubrication protection to a certain extent, but its effectiveness is relatively weak. Sample 3 exhibited a maximum coefficient of friction of 0.056 and an average coefficient of friction of 0.052, which is slightly higher than that of Sample 2. This indicates that the performance of the ZnS additive is marginally inferior to that of WS_2_. Nevertheless, ZnS can still serve as an effective lubricant additive for applications where lubrication performance requirements are not particularly stringent.

Sample 4 exhibited some fluctuation in its coefficient of friction during testing, with a maximum value of 0.052. The average coefficient of friction remained at 0.046, indicating relatively stable overall lubrication performance. This indicates that the T321 liquid additive can form an effective chemically adsorbed film at the friction interface, exhibiting anti-wear and extreme pressure properties. Compared to Sample 1, Sample 4 provides superior lubrication and protection. However, compared to Sample 2, Sample 4 exhibits greater fluctuations in its coefficient of friction, indicating slightly inferior stability during prolonged operation. This indicates that while Sample 4 provides reliable lubrication protection in the short term, there is room for improvement in terms of long-term stability and consistency.

2. Friction Coefficient–Time Analysis of Gel-Like Lubricating Grease for Wind Turbine Main Shaft Bearings Containing Hybrid Additives

**Figure 2:**
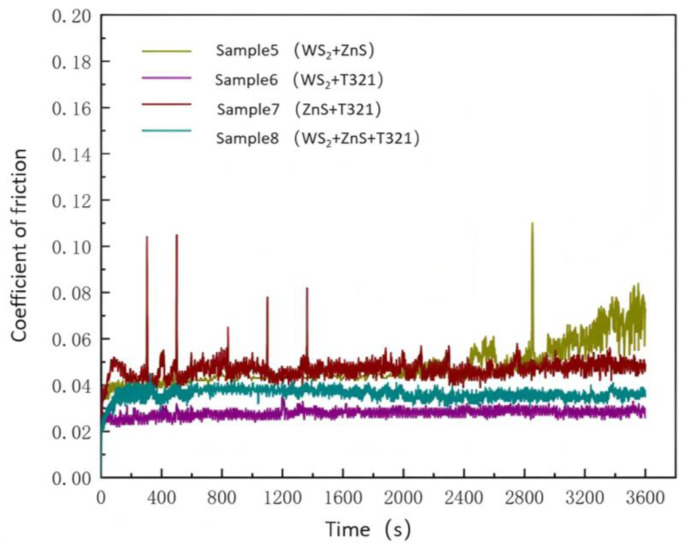
Friction Coefficient-Time Curve of Wind Turbine Main Bearing Grease with Mixed Additives.

The friction coefficient–time curves of gel-like lubricating grease for wind turbine main shaft bearings containing mixed additives, obtained using the MRS-10G lever-type four-ball friction and wear tester, are shown in Figure 2.

As shown in Figure 2, the tribological properties of Sample 5 exhibit distinct phased characteristics. During the initial running-in phase (0–2000 s), the friction coefficient remained stable at a low level of approximately 0.04, demonstrating excellent lubrication performance. However, after 2000 s, the friction coefficient began to rise and showed a distinct peak at 2805 s, indicating failure of the lubrication system. The underlying mechanism may be related to the interfacial behavior of the WS_2_/ZnS composite system: in the initial stage, the layered structure of WS_2_ can form an effective lubricating film under shear; however, the addition of ZnS may disrupt the ordered stacking and adsorption of WS_2_ sheets. Under continuous frictional heat and stress, this unstable composite interfacial film is prone to local collapse, leading to discontinuity of the lubricating film and a sharp increase in the friction coefficient. Sample 5 exhibited a maximum coefficient of friction of 0.193 and an average coefficient of friction of 0.058.

The coefficient of friction for Sample 6 remained relatively stable throughout the testing process, hovering around 0.03. This stability indicates a synergistic interaction between WS_2_ and T321, enabling the formation of a protective film on the friction surface that combines solid lubrication and chemical adsorption properties. This synergistic effect not only endows Sample 6 with outstanding lubricating properties but also enhances its thermal stability, enabling it to maintain a low coefficient of friction during prolonged operation. Sample 6 exhibited a maximum coefficient of friction of 0.034 and an average coefficient of friction of 0.024.

Sample 7 exhibited significant fluctuations in its coefficient of friction during the initial test phase (0–500 s) with relatively high values. This may be attributed to the lubricating film not yet being fully formed, indicating that the steel ball and Sample 7 were in the initial wear-in stage. As the test progressed, the coefficient of friction gradually stabilized within the range of 1500 to 3600 s, settling between 0.04 and 0.06. This stability indicates that after an initial break-in period, Sample 7 can form an effective lubricating film, thereby providing reliable anti-wear performance. Sample 7 exhibited a maximum coefficient of friction of 0.104 and an average coefficient of friction of 0.049.

Sample 8 exhibited a relatively stable coefficient of friction throughout the entire test process, with no significant fluctuations observed. During the initial phase of the test (0–400 s), the coefficient of friction for Sample 8 changed slowly and decreased, remaining between 0.03 and 0.04 as the test progressed. The maximum coefficient of friction is 0.044, with an average coefficient of friction of 0.037. This stable low coefficient of friction indicates that Sample 8 can form an effective lubricating film within a short time, demonstrating excellent lubricating properties and wear resistance.

**Figure 3:**
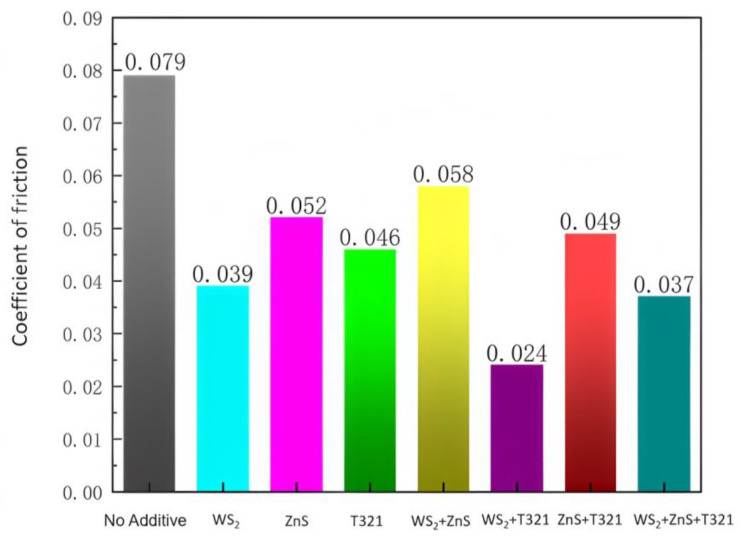
Average coefficient of friction of eight samples.

The average friction coefficients of the eight samples are shown in Figure 3.

As shown in Figure 3, the addition of different additives has a significant effect on the average coefficient of friction of the samples. Sample 1 served as the control group, exhibiting the highest average coefficient of friction at 0.079. Sample 2, Sample 3, and Sample 4 are samples with single additives, with average coefficients of friction of 0.039, 0.052, and 0.046 respectively, all lower than that of Sample 1. This indicates that each of these individual additives exhibits a certain degree of anti-wear effect, with Sample 2 demonstrating the most pronounced effect. This confirms that WS_2_ serves as an effective solid lubricant capable of reducing the coefficient of friction during the friction process. When the additives were combined, the average coefficients of friction for Samples 5 and 7 were 0.058 and 0.055, respectively. Both values were higher than that of Sample 2, which contained only WS_2_, but lower than that of Sample 1. This indicates that the combination of the two additives did not produce the expected synergistic effect. Instead, it may be due to chemical incompatibility between the additives or the formation of interfaces during friction that are detrimental to lubrication. Sample 6 exhibited the lowest average coefficient of friction at 0.024, significantly lower than other samples, indicating that the combination of WS_2_ and T321 delivers outstanding lubrication performance. This combination forms an effective protective film on the friction surface, thereby significantly reducing the coefficient of friction. This indicates that the rational selection and combination of additives in lubricant formulation design is crucial, as it can significantly enhance lubrication performance. Sample 8 exhibited an average coefficient of friction of 0.037, which, while higher than Sample 6, remained lower than most other samples, demonstrating excellent lubricating properties. This indicates that the competitive interactions among these three additives are more complex, resulting in lubrication performance that is inferior to the combination of two additives.

#### 2.1.2. Analysis of Steel Ball Scratch Diameter for Different Samples

Abrasion marks are a key subject of study in friction and wear testing, referring to the wear traces formed on the surfaces of friction pairs during the testing process. The size of the wear scar is a direct indicator of a material’s wear resistance. The smaller the scar, the stronger the material’s resistance to wear. Additionally, surface characteristics of friction marks—such as cracks, spalling, or oxide layers—can reveal the physical and chemical transformation mechanisms occurring within the material during the friction process. This study utilized a high-power microscope (4.5 × 0.5) to examine the wear scar morphology of steel balls after friction and wear testing, thereby evaluating the protective efficacy and wear mechanisms of eight samples.

1. Analysis of Steel Ball Wear Spot Diameter for Single Additive Samples

**Figure 4:**
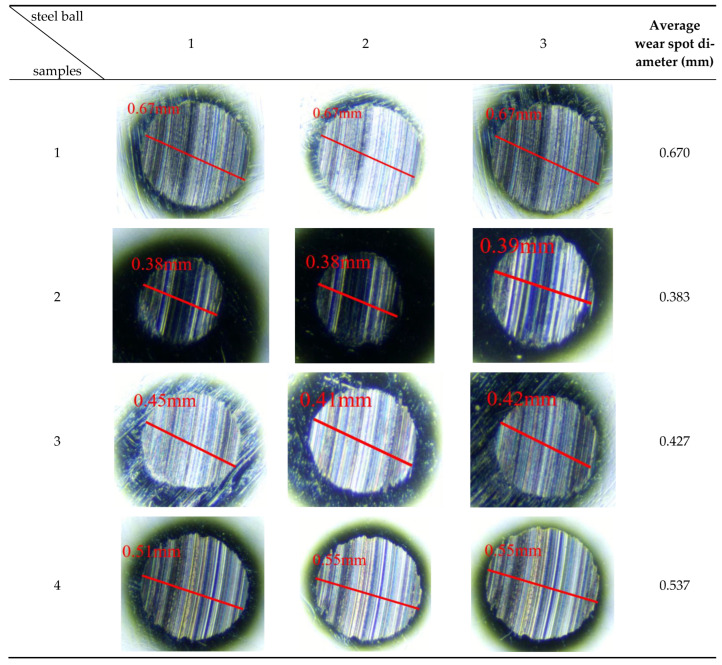
Morphology of wear spots on steel balls by single additive sample.

The microstructure of the wear scar on the steel ball caused by a single additive sample, as observed under a high-power microscope, is shown in Figure 4.

As shown in Figure 4, all three small ball wear scars in Sample 1 measure 0.67 mm in diameter, with an average diameter also of 0.67 mm. This indicates that the gel-like grease in Sample 1 exhibits poor lubricating performance during friction. It struggles to form an effective lubricating film on the steel ball surface, leading to increased direct metal-to-metal contact and accelerated wear of the steel balls. Although the wear spot diameters exhibit high consistency, the relatively large value of 0.67 mm indicates that Sample 1 demonstrates insufficient lubrication performance under extreme high temperatures, making it difficult to prevent direct metal-to-metal contact and wear. Additionally, the larger diameter of the wear scar suggests that Sample 1 may be prone to decomposition or loss under high-temperature conditions, resulting in reduced lubrication effectiveness. Therefore, Sample 1 has certain limitations in practical applications and requires further optimization to enhance its lubrication performance under complex operating conditions.

The diameters of the three small spheres containing Sample 2 were 0.38 mm, 0.38 mm, and 0.39 mm, respectively, with an average wear scar diameter of 0.383 mm. This result fully demonstrates that WS_2_ is an effective solid lubricant additive capable of forming a protective film on the surface of steel balls, reducing direct contact between them and thereby effectively minimizing wear. This protective coating not only delivers outstanding wear resistance but also maintains stability under extreme temperatures, resisting decomposition or loss. It provides robust assurance for the reliable operation of wind turbine main shaft bearings in high-temperature environments.

The diameters of the three small balls containing Sample 3 were 0.45 mm, 0.41 mm, and 0.42 mm, respectively, with an average wear scar diameter of 0.427 mm. This result indicates that ZnS exhibits a certain degree of anti-wear performance. Under high-temperature conditions, ZnS can form a protective film on the metal surface of steel balls, thereby reducing direct contact between the balls and minimizing wear. However, compared to WS_2_, the protective film formed by ZnS still falls short in terms of stability and anti-friction performance. This may be attributed to the close-packed hexagonal layered structure of ZnS, which exhibits low interlayer shear strength. Under extreme high-temperature conditions, the integrity of its protective film is prone to degradation.

The diameters of the three small spheres in Sample 4 were 0.51 mm, 0.55 mm, and 0.55 mm, respectively, with an average diameter of 0.537 mm. This indicates that the T321 additive possesses certain anti-friction properties, though its effectiveness is less pronounced than that of WS_2_ and ZnS. This may be because the protective film formed by T321 in high-temperature environments exhibits relatively poor stability and durability, making it more susceptible to damage during prolonged friction. Consequently, its protective effect on the steel ball surface is diminished.

2. Analysis of Steel Ball Wear Spot Diameter in Mixed Additive Samples

**Figure 5:**
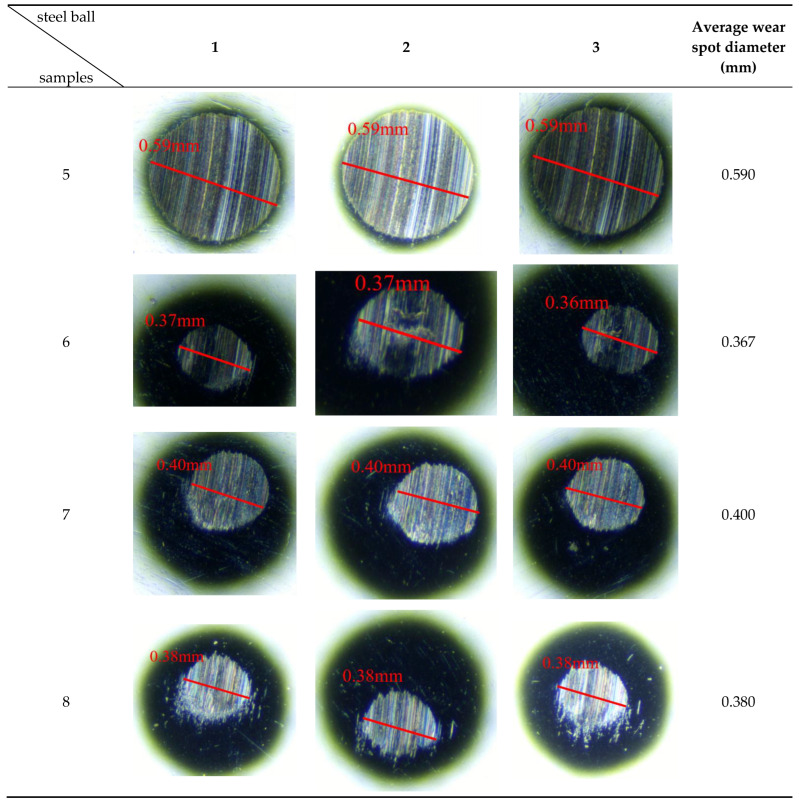
Morphology of abrasive spots on steel balls by mixed additive samples.

The micrograph of the mixed additive sample on the steel ball’s wear scar morphology, as shown in Figure 5.

As shown in Figure 5, the diameters of the three balls containing Sample 5 are all 0.59 mm, 0.59 mm, and 0.59 mm, respectively, with an average template diameter of 0.59 mm. This result indicates that the WS_2_ and ZnS mixed additives exhibit a negative synergistic effect. At high temperatures, WS_2_ forms a stable lubricating film on friction surfaces due to its layered structure, demonstrating excellent anti-friction and anti-wear properties. However, when used in combination, ZnS may compromise the integrity of the WS_2_ lubricating film, leading to disruption of its continuity during friction. This prevents WS_2_ from fully realizing its anti-friction advantages.

The diameters of the three small balls in Sample 6 were 0.37 mm, 0.37 mm, and 0.36 mm, respectively, with an average template diameter of 0.367 mm. This result indicates that Sample 6 demonstrates outstanding performance in reducing friction and wear, outperforming other samples and exhibiting a significant synergistic effect. At elevated temperatures, WS_2_ functions as a solid lubricant to form a dense and stable protective film on metal surfaces, effectively reducing direct contact between steel balls. Meanwhile, T321 enhances the lubrication effect by improving the flowability and dispersibility of gel-like grease, ensuring uniform coverage of WS_2_ across metal surfaces.

The diameters of the three small balls in Sample 7 are all 0.40 mm, 0.40 mm, and 0.40 mm, respectively, with an average template diameter of 0.40 mm. This indicates that Sample 7 has a certain effect in reducing friction and wear. However, compared to Sample 6, Sample 7’s overall performance was not satisfactory. This difference may be attributable to WS_2_’s unique layered structure and superior lubricating properties, which render it more effective than ZnS in reducing the coefficient of friction at the contact surface. Therefore, although Sample 7 demonstrates certain application potential, further exploration and optimization are still required for practical engineering applications.

The three small spheres containing Sample 8 had spot diameters of 0.38 mm, 0.38 mm, and 0.38 mm, respectively, with an average spot diameter of 0.38 mm. This result indicates that Sample 8 demonstrated excellent performance in reducing the diameter of scuff marks, with properties comparable to Sample 6 and superior to the other samples. However, the higher additive content in Sample 8, while improving lubrication performance, also revealed some potential issues. Interactions between multiple additives may lead to insufficient stability of the lubricating film. Particularly under high-temperature conditions, the continuity and integrity of the lubricating film may be disrupted, thereby compromising its long-term reliability. Furthermore, excessive use of additives not only increases production costs but may also impose unnecessary environmental burdens.

**Figure 6:**
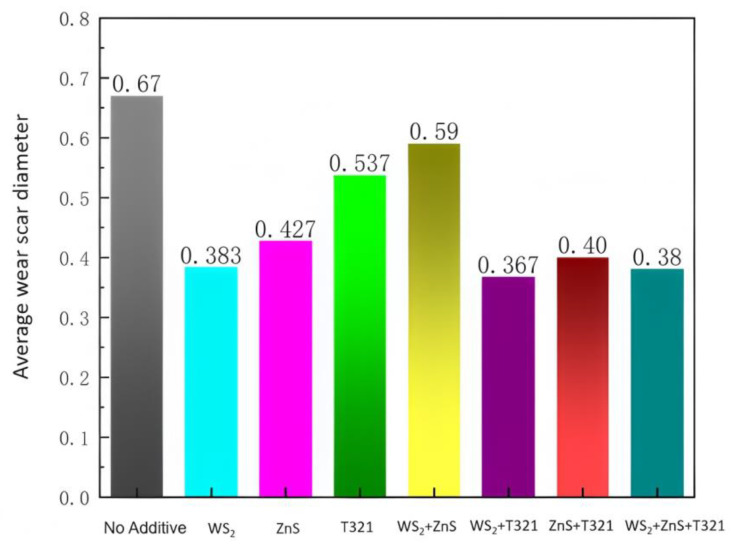
Average template diameter for eight samples.

The average abrasion spot diameter for the eight samples is shown in Figure 6. The underlying cause of the differences in tribological performance lies in the distinct action mechanisms of the various additives. For the optimal WS_2_/T321 system, its exceptional friction-reducing and anti-wear performance stems from a synergistic effect between physical lubrication and tribochemical reactions. Tungsten disulfide (WS_2_) possesses a typical layered crystal structure where the interlayers are bound by relatively weak van der Waals forces. Under frictional shear stress, these layers slide easily, forming a solid lubricating film with low shear strength on the contact surface. This film directly separates the friction pair, effectively reducing mechanical friction. Simultaneously, sulfurized isobutylene (T321), as an active sulfur-containing additive, can undergo tribochemical reactions with the steel surface under the high temperature and pressure generated by friction, producing lubricious sulfide reaction films such as FeS. This reaction film not only further reduces the surface shear strength but also repairs micro-damage. The synergy between the two lies in the fact that the layered physical film of WS_2_ provides initial protection and a stable low-friction interface, creating favorable conditions for T321 to form a uniform reaction film. In turn, the chemical film generated by T321 fills potential coverage defects in the physical film. Together, they construct a more continuous and stable composite lubrication interface. This constitutes the intrinsic mechanism behind its simultaneously lowest friction coefficient and wear scar diameter and provides a key theoretical basis for its long-term lubrication performance under the high-temperature and heavy-load conditions of wind turbine bearings.

Among the eight samples, Sample 6 (WS_2_/T321 composite) demonstrated the most outstanding performance in both coefficient of friction and wear scar diameter. The average abrasion spot diameter measured from three replicate tests was 0.367 ± 0.015 mm, significantly smaller than that of other samples (e.g., compared to Sample 1, *p* < 0.01). This result is highly consistent with the conclusions from the coefficient of friction tests, collectively indicating that the compound of WS_2_ and T321 can most effectively reduce friction and wear in the contact pair under the experimental conditions. This indicates that the formulation holds the greatest potential for enhancing the high-temperature anti-wear properties of gel-type greases, making it the preferred candidate for subsequent validation closer to engineering applications, such as bearing bench testing.

### 2.2. Optimization Analysis of Rheological Properties of Gel-like Lubricating Grease for Wind Turbine Main Shaft Bearings

#### 2.2.1. Analysis of Flow Characteristics of Different Samples

**Figure 7:**
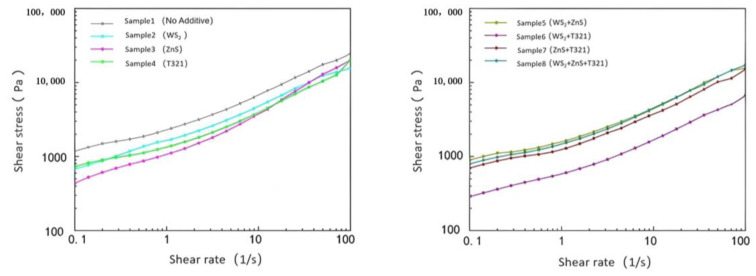
Variations in shear stress with shear rate for eight samples at −20 °C.

The flow characteristics of gel-type grease directly affect the lubrication effectiveness, heat dissipation performance, and fatigue life of spindle bearings. Particularly under extreme temperatures, gel-type grease may experience oil separation, softening, or hardening, leading to lubrication failure and increased friction and wear on bearing components. Through in-depth investigation of the flow characteristics of gel-like grease, a comprehensive understanding of its behavior under external forces can be achieved. Figure 7 shows the variation in shear stress with shear rate for eight samples at −20 °C.

(1) Analysis of Flow Characteristics of Eight Samples at −20 °C

As shown in Figure 7, the shear stress of the eight samples increases with increasing shear rate and exhibits a nonlinear growth trend. Sample 1 exhibits typical non-Newtonian fluid characteristics. It demonstrates a certain degree of flow behavior at low shear rates, but its growth trend flattens out at medium to high shear rates, indicating a weakening of its shear-thickening effect. This indicates that Sample 1 performs well at low to moderate shear rates, but its flow properties become constrained at high shear rates. 

The shear stress of Sample 2 increases with shear rate, though its value remains slightly lower than that of Sample 1. This indicates that the WS_2_ additive has taken effect in Sample 2, effectively improving the material’s flow characteristics and shear thinning properties. This may be related to the oriented arrangement of WS_2_ under shear stress. As shear rate increases, the layered structure of WS_2_ additives undergoes orientation under shear stress, forming a more regular structure. This effectively improves the material’s flow characteristics and shear thinning properties. Sample 3 exhibits lower shear stress at low shear rates but demonstrates a more pronounced stress increase at medium to high shear rates, indicating that its thickener fiber network maintains a certain degree of structural integrity under high shear conditions. This characteristic may be related to the ZnS in Sample 3, which can form a stable lubricating film at low temperatures but may experience a slowdown in stress growth at high shear rates due to partial rupture of the fiber network. Sample 4 exhibited a steady increase in shear stress without any significant stress spikes or drops, indicating that its internal structure remains stable under high shear conditions. This may be due to intermolecular forces or chemical bonding within the T321 structure, enabling the fiber network to maintain a certain level of strength under shear stress. This prevents structural collapse or oil separation in the gel-like lubricant.

**Figure 8:**
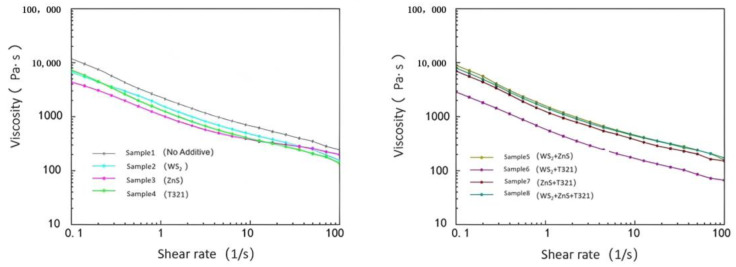
Variation in viscosity with shear rate for eight samples at −20 °C.

Comparative analysis revealed that Sample 6 demonstrated the most outstanding performance among the eight samples. Its shear stress remained relatively low across the entire shear rate range, with a stable growth slope, indicating exceptional shear resistance and excellent flow properties. This outstanding performance can be attributed to the synergistic interaction between WS_2_ and T321: WS_2_, acting as a solid lubricant, forms a stable protective film on friction surfaces, while T321 further enhances the strength and durability of the lubricating film through chemical adsorption. This dual-protection mechanism enables Sample 6 to maintain low friction loss even under high shear rates, making it suitable for high-speed, high-load extreme operating conditions. In contrast, although Sample 8 contained three additives, its performance improvement did not meet expectations. This may be due to the complex interactions among multiple additives, preventing their effects from fully combining. Next, we will explore the viscosity changes of eight samples with respect to shear rate, as shown in Figure 8. 

As shown in Figure 8, under extreme low-temperature conditions of −20 °C, the viscosity of all eight samples decreased with increasing shear rate. Sample 1 exhibits relatively high viscosity at all shear rates, and its viscosity decreases at a relatively gentle rate as shear rate increases. This indicates that Sample 1 exhibits only a limited reduction in viscosity under high shear rates, which may result in suboptimal lubrication performance in low-temperature environments. The high viscosity of Sample 1 may be related to the low-temperature performance of its base oil and thickener. At −20 °C, the molecular motion of Sample 1’s base oil is restricted, leading to a significant increase in the overall viscosity of the gel-type grease. The thickener’s fiber network may become more tightly packed at low temperatures, further limiting the grease’s flowability.

Comparative analysis reveals that Sample 6 exhibits a smoother viscosity–shear rate curve with the most pronounced viscosity reduction. This indicates that Sample 6 demonstrates superior flow characteristics under high shear rates, enabling more effective friction and wear reduction to deliver enhanced lubrication performance. However, although Sample 8 contained three additives, its viscosity decrease was not as pronounced as that of Sample 6.

In summary, under extreme low-temperature conditions of −20 °C, Sample 6 exhibited the best flow characteristics. Its viscosity decreases significantly with increasing shear rate, enabling Sample 6 to maintain low friction loss and stable lubrication performance even under high shear conditions. This characteristic is particularly crucial for wind turbine main shaft bearings operating under extreme temperatures, as the fluidity of gel-like grease in low-temperature environments directly impacts the bearings’ start-up performance and operational efficiency.

(2) Analysis of Flow Characteristics of Eight Samples at 80 °C

**Figure 9:**
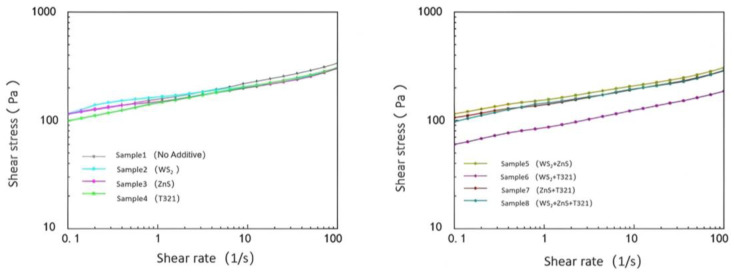
Variation in shear stress with shear rate for eight samples at 80 °C.

As shown in Figure 9, at the extreme high temperature of 80 °C, the shear stress of all eight samples increased with increasing shear rate. However, differences were observed in the rate of shear stress increase and the final shear stress values achieved among the different samples. Comparative analysis reveals that although Sample 1 exhibits favorable shear stress at low shear rates, its limited capacity to increase shear stress at high shear rates indicates certain limitations. This indicates that Sample 1 exhibits favorable lubricating properties at low shear rates. However, at high shear rates, its shear stress increases rapidly, suggesting limited stability under high-shear conditions. This may be due to the base oil in Sample 1 being unable to effectively maintain the integrity of the lubricating film under high shear rates, leading to increased friction and consequently a significant rise in shear stress. 

In contrast, Sample 6 exhibited lower shear stress and a more gradual increase trend. Sample 6’s low shear stress characteristics enable it to effectively reduce friction and wear at high shear rates, thereby delivering more stable and reliable lubrication performance. This property not only helps minimize energy loss during bearing operation but also reduces temperature rise caused by frictional heat, preventing gel-type greases from failing due to high-temperature oxidation or oil separation.

**Figure 10:**
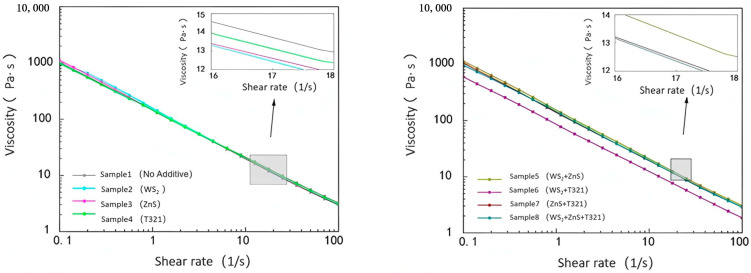
Viscosity variation with shear rate for eight samples at 80 °C.

In summary, Sample 6 demonstrates excellent stability and adaptability under extreme high-temperature conditions of 80 °C. This characteristic enables Sample 6 to effectively reduce friction and wear at high shear rates, thereby providing more stable and reliable lubrication protection. The viscosity of the eight samples as a function of shear rate will be analyzed next, as shown in Figure 10. 

As shown in Figure 10, under extreme high-temperature conditions of 80 °C, the viscosity of all eight samples decreased with increasing shear rate, exhibiting a clear negative correlation trend. This phenomenon reveals that the samples generally exhibit shear thinning behavior under high-temperature conditions. Sample 6 stood out among these samples, demonstrating excellent flow stability due to its low viscosity and significant decrease in viscosity with increasing shear rate. Even under the dual challenges of high temperature and high shear rate, Sample 6 maintained stable lubricating performance, effectively preventing lubrication failure caused by rapid viscosity decline. This characteristic enables it to provide reliable lubrication protection under high-temperature conditions, reducing wear between bearing components. The shear stress changes of the eight samples under extreme temperatures are shown in Table 1.

#### 2.2.2. Analysis of Viscosity–Temperature Characteristics for Different Samples

The viscosity–temperature characteristics of gel-like grease refer to the pattern of viscosity variation with temperature, which directly affects its flowability and lubricating performance. An ideal gel-type grease should maintain good fluidity at low temperatures to ensure smooth startup; at high temperatures, it must possess sufficient viscosity to maintain the stability of the lubricating film, preventing film breakdown and accelerated wear. Therefore, studying the viscosity–temperature characteristics of gel-like greases aids in selecting suitable formulations and optimizing lubrication performance under various operating conditions.

(1) Analysis of the Viscosity–Temperature Characteristics of Eight Samples at −20 to 30 °C

**Figure 11:**
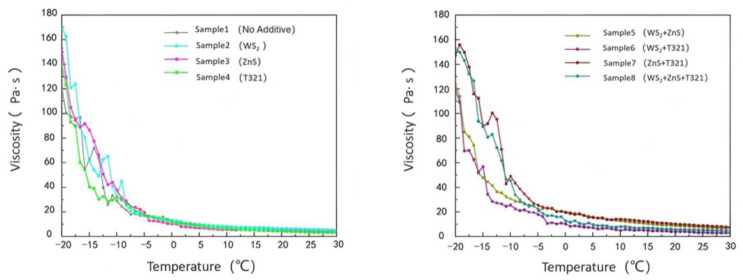
Viscosity variation with temperature for eight samples at −20~30 °C.

As shown in Figure 11, at −20 °C, Sample 1 exhibited a viscosity of 121.76 Pa·s. This indicates that under low-temperature conditions, the sample demonstrates extremely poor flowability and exhibits high internal friction resistance. At temperatures ranging from −20 °C to −7.5 °C, all test samples exhibited high viscosity and a degree of instability, with some samples even showing an upward trend in viscosity. This phenomenon can be attributed to the slowed molecular motion of gel-like grease in low-temperature environments, which increases intermolecular friction resistance and consequently raises viscosity. However, as the temperature gradually rose to 30 °C, the viscosity of all samples exhibited a significant downward trend. This is because the increase in temperature promotes thermal motion between molecules, reduces intermolecular forces, and consequently enhances the sample’s fluidity.

At −20 °C, the viscosities of Samples 2 and 3 were both higher than that of Sample 1, which was as expected. Since both WS_2_ and ZnS are powdered solid particles, the addition of these solid particles increases the complexity within the sample. This leads to more restricted intermolecular motion at low temperatures, resulting in increased internal friction resistance and consequently elevated viscosity. However, as the temperature gradually increased, the viscosity change trends of Samples 2 and 3 differed from that of Sample 1. This is because solid particles can be more effectively dispersed throughout the sample, forming a stable suspension that provides additional support and lubrication. This reduces intermolecular forces while enhancing the wear resistance of the gel-based grease. T321 is a liquid additive with excellent oil solubility that rapidly forms a polymeric protective layer on metal surfaces. This enhances the flow properties of Sample 4 at low temperatures, improving its fluidity and lubricity under cold conditions. 

**Figure 12:**
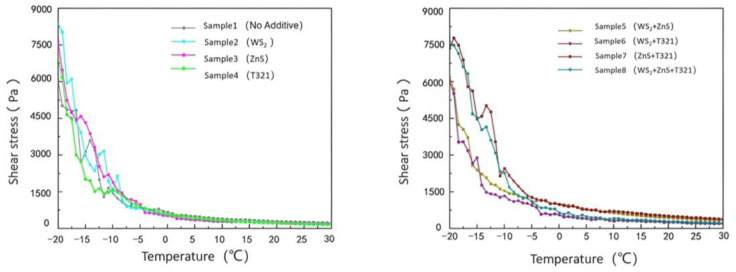
Variation in shear stress with temperature for eight samples at −20~30 °C.

Comparative analysis reveals that Sample 6 exhibits a smooth and moderate downward trend in its viscosity change curve. This phenomenon indicates that the internal structure of Sample 6 is relatively stable, with solid particles and liquid additives forming an efficient synergistic effect to construct a more effective lubrication network. This lubrication network not only reduces internal resistance within the gel-like grease at low temperatures, making the sample easier to flow in cold conditions, but also better adapts to temperature changes. To gain a deeper understanding of the viscosity–temperature characteristics of the eight samples, we next analyze the variation in shear stress with temperature, as shown in Figure 12. 

As shown in Figure 12, the shear stress of all eight samples exhibits an overall decreasing trend with increasing temperature, with variations similar to those observed in their viscosity changes with temperature. Sample 6 exhibits favorable performance within the temperature range of −20 to 30 °C, maintaining relatively low shear stress values. This indicates that Sample 6 not only effectively reduces starting torque within this temperature range, enhancing equipment startup performance and operational efficiency, but also rapidly responds to the lubrication demands of mechanical components.

(2) Analysis of the viscosity–temperature characteristics of eight samples at 30–80 °C

**Figure 13:**
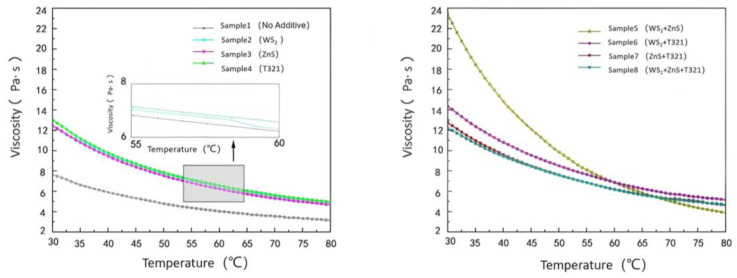
Viscosity variation with temperature for eight samples from 30~80 °C.

As shown in Figure 13, the viscosity of all eight samples decreases with increasing temperature. Among them, Sample 1 consistently maintains a low viscosity level, and its tendency toward low viscosity under high-temperature conditions becomes increasingly pronounced. Sample 1 exhibits relatively low viscosity at 30 °C, and its viscosity decreases further as temperature rises, indicating its inability to maintain sufficient oil film thickness at elevated temperatures. This condition of excessively low viscosity may cause the lubricating film to break down, increasing direct metal-to-metal contact and thereby accelerating wear and friction. Therefore, the primary shortcoming of Sample 1 lies in its excessively low viscosity under high-temperature conditions, which leads to diminished lubricating performance and limits its applicability in high-temperature operating environments. 

Comparative analysis reveals that Sample 5 exhibits higher viscosity at 30 °C. This is primarily attributed to the inclusion of WS_2_ and ZnS additives, both in powdered particle form, which increase the internal friction within Sample 5, thereby significantly enhancing its viscosity. This high viscosity results in poor fluidity for Sample 5, increasing the risk of friction and wear during the startup of bearing components. Meanwhile, although the viscosity of Sample 5 decreases with increasing temperature, the rate of decrease is significant. This indicates that its lubricating performance is unstable under temperature variations, making it difficult to effectively counteract the effects of temperature changes and thus unable to provide reliable lubrication protection. In contrast, Sample 6 performed quite well. It exhibits moderate viscosity at 30 °C, ensuring excellent fluidity and effectively reducing friction and wear on mechanical components during startup. Sample 6 exhibits a steady decrease in viscosity with increasing temperature. Even at 80 °C, it maintains adequate viscosity, demonstrating excellent high-temperature stability. It can sustain sufficient lubricating film thickness in high-temperature environments, providing continuous and effective lubrication protection.

**Figure 14:**
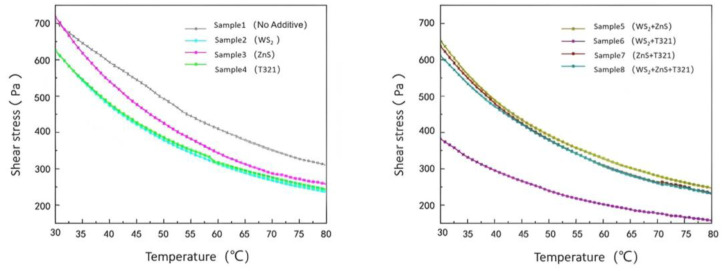
Variation in shear stress with temperature for eight samples at 30~80 °C.

Next, we will investigate the temperature dependence of shear stress for eight samples, as shown in Figure 14.

As shown in Figure 14, the shear stress of the eight samples gradually decreases with increasing temperature. Sample 1 exhibits significantly higher shear stress at 30 °C compared to other samples, indicating poor fluidity. This impedes the formation of an effective lubricating film during the startup phase, leading to increased friction resistance. Consequently, it exacerbates energy loss, localized overheating, and wear, thereby compromising equipment efficiency and service life. As temperature increases, the shear stress of Sample 1 decreases but remains higher than that of other samples, indicating its poor viscosity–temperature characteristics and inability to maintain stable lubrication performance across a wide temperature range. 

In contrast, Sample 6 exhibited significantly lower shear stress at 30 °C compared to other samples, demonstrating excellent low-temperature flow and lubrication properties. This low shear stress characteristic enables it to rapidly form a uniform and stable lubricating film during the initial start-up phase of the bearing. This effectively reduces friction and wear during startup, providing the bearing with excellent start-up protection. As temperature increases, the shear stress of Sample 6 exhibits a steady decline, demonstrating excellent viscosity–temperature characteristics. This property enables it to maintain stable lubrication performance across a wide temperature range.

The viscosity changes of eight samples under extreme temperatures at −20 to 30 °C and 30 to 80 °C are shown in Table 2.

The superior performance of the WS_2_/T321 composite system compared to single additives stems from the formation of a reinforced lubricating film at the friction interface through synergistic complementarity between physical adsorption and chemical reactions. Pure WS_2_ relies on the physical sliding of its layered structure to reduce friction, but under high temperature and shear, a purely physically adsorbed film may fail due to mechanical disruption or desorption. Pure T321, on the other hand, depends on harsh tribochemical reactions to generate a protective film, whose formation efficiency and uniformity are limited by the initial contact conditions. In the composite system, the layered WS_2_ particles first spread rapidly via physical adsorption, forming an initial, low-shear-strength separating film in the contact zone. This film not only directly reduces friction and temperature rise but, more critically, provides a milder and more stable interfacial environment for the tribochemical reactions of T321. In this environment, T321 can react with the metal surface more efficiently and uniformly, generating chemically bonded reaction films such as FeS. This chemical reaction film, in turn, “anchors” and consolidates the WS_2_ physical film, preventing its removal under prolonged shear, thereby constructing a dynamically stable, multi-layer composite lubrication interface characterized by “the physical film laying the foundation for the chemical reaction, and the chemical film anchoring the physical film”. This synergistic mechanism enables the composite system to maintain the integrity and effectiveness of the lubricating film over a wide temperature range and extended duration, resulting in a “1 + 1 > 2” enhancement effect in both tribological and rheological performance.

## 3. Conclusions

### 3.1. Conclusions

This study conducted a comprehensive evaluation of composite lithium-based gel greases containing different sulfur additives through systematic tribological and rheological testing. The main conclusions are as follows:

1. The composite system of WS_2_ and T321 (Sample 6) exhibits outstanding synergistic performance. In terms of tribology, both its average coefficient of friction (0.024 ± 0.003) and average wear scar diameter (0.367 ± 0.015 mm) were the lowest. Specifically, compared to the base grease (Sample 1), this system reduced the average friction coefficient by approximately 69.6% (from 0.079 to 0.024) and decreased the average wear scar diameter by about 45.2% (from 0.670 mm to 0.367 mm). At a fixed addition level (0.5 wt.%), all sulfur-containing additives improved the high-temperature (80 °C) anti-friction and wear resistance of the base oil to some extent. The advantage of this combination lies in that the layered structure of WS_2_ provides a physical lubricating film with low shear strength, while T321 generates a chemisorbed film through tribochemical reactions; the two act synergistically to form a more stable and durable composite lubrication interface.
2. In rheology, the WS_2_ and T321 blend system exhibits the highest shear stability at 80 °C (with a gradual increase in shear stress) and demonstrates optimal viscosity–temperature characteristics and flow properties across a broad temperature range from −20 °C to 80 °C. This ensures its lubricant performance capability under extreme high and low temperatures. Rheological testing further confirmed that the modified lipids retained the base lipid’s non-Newtonian fluid properties and colloidal stability, demonstrating the fundamental feasibility of additive modification.
3. Based on comprehensive tribological and rheological data, the WS_2_/T321 compounding system was identified as the optimal candidate formulation under the laboratory testing conditions established in this study. This work not only identified high-performance additive combinations but also provided theoretical support through rheological analysis for their pumpability, retention, and operational consistency in actual bearings. It has laid a solid experimental and theoretical foundation for the subsequent development of wind turbine bearing greases designed for extreme operating conditions. Therefore, the lubricant formulation proposed in this study is expected to directly enhance the operational reliability and extend the service life of wind turbine spindle bearings under harsh operating conditions by significantly reducing friction and wear while maintaining stable lubrication performance across both high and low temperatures.

### 3.2. Outlooks

This study conducted preliminary screening and performance characterization of additive formulations based on laboratory-standard tribological and rheological testing. To advance the research findings toward engineering applications with greater confidence, subsequent work may be pursued in the following areas:

1. Microscopic Elucidation of Lubrication and Wear Mechanisms: Current understanding of the synergistic effect between WS_2_ and T321 remains based on macroscopic performance inferences. Subsequent studies should employ surface analysis techniques such as scanning electron microscopy (SEM), focused ion beam (FIB), and X-ray photoelectron spectroscopy (XPS) to conduct detailed analyses of the microstructure, elemental distribution, and chemical states on friction surfaces. This approach will directly reveal the formation processes, structures, and mechanisms of action for friction chemical reaction films and solid lubrication layers at the atomic/molecular level, providing precise guidance for formulation optimization.
2. Bench testing simulating real operating conditions: Four-ball tests and rheological analyses serve as effective screening tools, yet they fall short of replicating the actual complex operating conditions of wind turbine main shaft bearings—such as alternating loads, combined sliding and rolling motion, and grease degradation after prolonged operation. The core focus of the next phase of research is to conduct long-term, dynamic durability, reliability, and service life evaluations of the selected optimal formulation (WS_2_/T321) on a dedicated test rig simulating wind turbine bearing operating conditions. This serves as the essential bridge connecting laboratory research with engineering applications.
3. Full Life Cycle Performance and Compatibility Assessment: Future research may further evaluate the performance of this modified grease in terms of long-term oxidation, thermal aging, and compatibility with bearing seal materials. Simultaneously, the temperature range can be extended to lower extremes (such as −40 °C or below), and its performance stability in contaminated environments (water, dust) should be investigated to establish a comprehensive evaluation system for its applicability throughout its entire lifecycle.

## 4. Materials and Methods

### 4.1. Test Equipment and Materials

#### 4.1.1. MRS-10G Lever-Type Four-Ball Friction and Wear Tester

**Figure 15:**
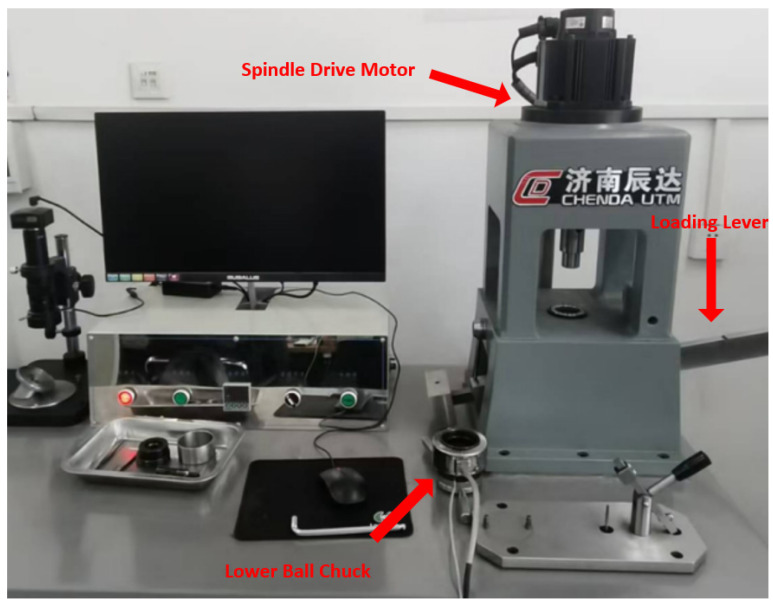
MRS-10G Lever Type Four-Ball Friction and Wear Tester.

The MRS-10G Lever-Type Four-Ball Friction and Wear Tester (Jinan Chenda, Jinan, China) (as shown in Figure 15) is an advanced laboratory testing device manufactured by Jinan Chenda, widely used in the performance evaluation of lubricants. This friction and wear testing machine utilizes a three-phase motor with a power rating of 1.5 kW. The spindle speed ranges from 200 to 3000 rpm, while the axial load capacity spans from 98 to 9800 N. The oil cup heating temperature can reach up to 250 °C. Its design is based on the classic four-ball friction and wear test principle. By simulating the sliding friction process in actual mechanical components, it qualitatively collects parameters such as friction force and coefficient of friction to evaluate the load-carrying capacity and anti-wear properties of lubricants. Additionally, this testing machine’s capabilities extend beyond evaluating the mechanical properties of materials. It can also examine the wear morphology and diameter of steel balls through its integrated microscope, with a precision of 0.01 mm, to investigate wear characteristics and assess their abrasion resistance.

#### 4.1.2. Anton Paar MCR302 Rotational Rheometer

**Figure 16:**
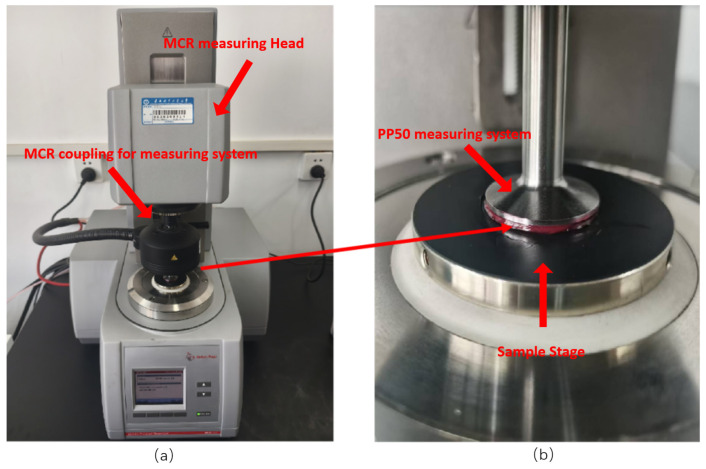
(**a**) MCR302 Rotational Rheometer. (**b**) Rheometer parallel-plate measuring geometry.

The Anton Paar MCR302 Rotational Rheometer (Anton Paar, Graz, Austria) is an advanced laboratory instrument (as shown in Figure 16) primarily used to measure rheological parameters—such as shear stress, shear rate, viscosity, and loss factor—of various materials including liquids and soft solids, thereby evaluating their rheological properties. This instrument features a broad testing range, with rotational speeds between 10^−6^ and 200 r/min, torque measurements from 10 μN·m to 0.2 N·m, and normal force measurements from 0.001 to 50 N. Its built-in RheoCompass application (Version 1.30, Anton Paar, Graz, Austria) is an advanced rheological testing and analysis software developed by Anton Paar. It breaks down complex rheological testing into multiple test items and modules, including test procedures, graphs, data, displays, analysis, and reporting of wear scar patterns. This software possesses the capability to handle complex rheological models and predict rheological properties. 

#### 4.1.3. Selection and Optimization of Gel-Type Grease

**Figure 17:**
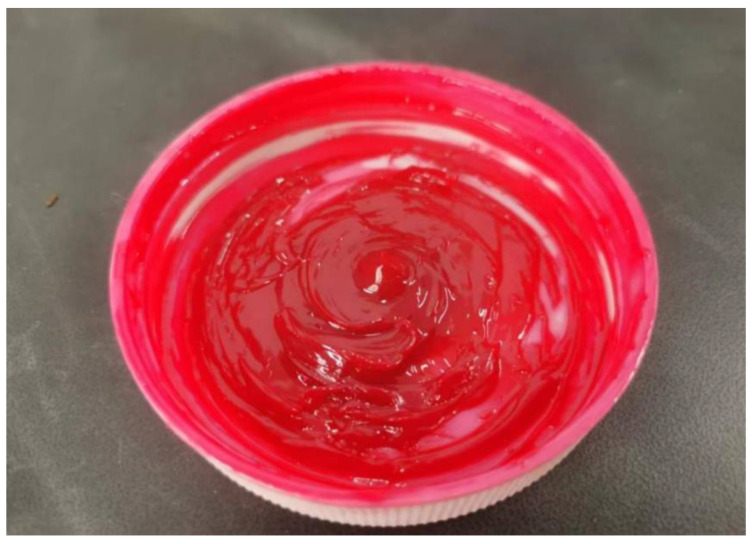
Mobil SHC 460WT Lithium Complex Grease.

Under extreme temperatures, selecting the appropriate lubricant type is critical for the operation of wind turbine main shaft bearings [49]. Wind turbine units are often located in environments such as deserts, mountain wind corridors, and coastal islands [50]. Consequently, the main shaft bearings must not only withstand irregular loads caused by wind forces but also endure harsh conditions including extremely low temperatures, high humidity, salt spray, and dust. These factors not only increase the likelihood of bearing wear and corrosion but also interfere with the functionality of gel-based lubricants, potentially leading to premature bearing failure. Therefore, selecting appropriate lubricants is essential for ensuring the durability of wind turbine spindle bearings and maintaining high operational efficiency. In this study, Mobil SHC 460WT lithium complex grease (Zhengzhou Aote Technology Co., Zhengzhou, China) was selected as the base lubricant. It is formulated with a high-quality ISO VG 460 synthetic base oil (Zhengzhou Aote Technology Co., Zhengzhou, China) and has an NLGI 1.5 consistency grade, which provides a balance of good pumpability and adequate structural stability. Its specified extreme-pressure and anti-wear performance can reduce friction and wear under typical operating conditions. Quantitative comparison with the modified greases reveals the effectiveness of the optimized formulation: the best-performing WS_2_/T321 composite grease reduced the average friction coefficient by approximately 69.6% and decreased the average wear-scar diameter by about 45.2% relative to the base grease. This clear quantitative improvement underscores the potential of additive modification to significantly enhance lubrication performance for demanding wind-turbine applications. This gel-like grease offers excellent low-temperature fluidity, ensuring reliable starting performance in severe cold conditions. It also features exceptional oxidation resistance, delaying oil degradation and guaranteeing long-term stable operation. Its high-temperature resistance enables it to maintain excellent lubrication performance in high-temperature environments while effectively resisting corrosion, protecting wind turbine main shaft bearings from environmental impacts. Due to its superior comprehensive performance, it has become the ideal choice for lubricating wind turbine main shaft bearings. Figure 17 shows Mobil SHC 460WT Complex Lithium Gel Grease, and Table 3 lists the performance parameters of Mobil SHC 460WT Complex Lithium Gel Grease.

Although Mobil SHC 460WT Compound Lithium-Based Gel Grease performs well in numerous applications, it still exhibits certain shortcomings when used long-term in wind turbine main shaft bearings under extreme temperatures [51]. First, under the influence of adverse environmental factors such as large temperature differentials, severe fluctuations, high humidity, dust, and salt spray, the aging and performance degradation of this gel-like grease are significantly accelerated. These external conditions not only accelerate the oxidation process of gel-type grease but may also cause changes in its viscosity and a decline in its corrosion resistance, thereby weakening its effective protection of the bearings [52]. Additionally, the continuous variation in wind turbine load is also a significant factor. Prolonged high-load operation can cause gel-type grease to degrade prematurely at high temperatures, reducing its lubricating effectiveness and consequently shortening the bearing’s service life. Especially under extreme low-temperature conditions, gel-like grease may become excessively viscous, failing to provide sufficient fluidity to ensure adequate lubrication and thereby increasing the risk of equipment wear [53].

The combined effects of these issues have resulted in Mobil SHC 460WT Compound Lithium-Based Gel Grease performing below expectations in practical applications, particularly exhibiting significant shortcomings when subjected to extreme temperature fluctuations and complex operating conditions. Therefore, to enhance the stability and long-term performance of Mobil SHC 460WT Complex Lithium-Based Gel Grease under extreme temperatures, optimizing its formulation is of paramount importance. By optimizing the formulation, the extreme pressure, flow, and anti-friction wear properties of gel-type grease can be enhanced, ensuring it continues to provide outstanding lubrication protection for wind turbine main shaft bearings under various harsh conditions. This extends equipment service life and improves operational efficiency [54].

In recent years, the performance optimization of gel-like bearing greases has become one of the research hotspots in the field of lubricants. Under extreme temperature conditions, the performance stability and efficiency of traditional gel-based greases face challenges, which directly impact the operational efficiency and service life of wind turbines. The premature degradation of traditional gel-like greases in high-temperature environments is primarily attributed to accelerated oxidation and thermal breakdown of the base oil and thickener, which compromises the structural integrity of the lubricating film. At low temperatures, the pronounced increase in viscosity stems from restricted molecular motion and the strengthening of the thickener’s network, hindering effective flow to the contact interfaces. These inherent limitations in conventional formulations underscore the critical need for advanced lubricants capable of maintaining thermal stability and controlled viscosity across the extreme operating temperature range of wind turbine bearings. Optimizing grease composition with tailored additives is therefore essential to ensure reliable lubrication, minimize wear, and enhance the overall durability of the transmission system.

To address these challenges, researchers have begun exploring various strategies to optimize the performance of gel-like lubricants for bearings. One significant approach involves enhancing the thermal stability, mechanical stability, and oxidation resistance of gel-like lubricants through the use of different types of additives and high-performance base oils. For example, additives such as anti-wear agents, antioxidants, and extreme pressure additives can significantly enhance the performance of gel-type greases under extreme conditions. However, excessive additives not only increase production costs but may also introduce unnecessary chemical components, compromising the stability and environmental friendliness of the emulsifier. To maintain or enhance the performance of spindle gel-type lubricants while reducing additives, researchers began seeking more efficient and environmentally friendly additives.

**Figure 18:**
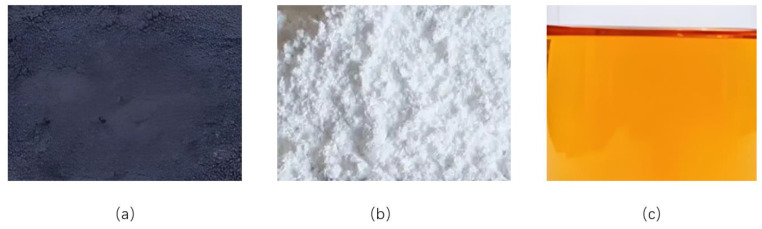
Three different additives: (**a**) Tungsten disulfide; (**b**) Zinc sulfide; (**c**) Isobutylene sulfide.

Sulfide additives play a significant role in the lubricant field due to their unique chemical characteristics and physical properties. At low temperatures, it maintains the fluidity of gel-based grease, reduces friction resistance during cold starts, lowers energy consumption, and protects equipment. Under high-temperature conditions, sulfides exhibit exceptional thermal stability, effectively preventing the oxidation and degradation of gel-based lubricants to ensure long-term, high-efficiency operation. Sulfides also form a robust yet flexible protective film on metal contact surfaces, providing additional load-bearing capacity under high stress, absorbing vibrations, reducing noise, and enhancing operational smoothness. Additionally, sulfides exhibit excellent biodegradability, making them environmentally friendly and consistent with sustainable development requirements. Therefore, sulfide additives represent an ideal choice for enhancing lubricant performance in terms of both technical properties and environmental protection. The three additives selected for this study are WS_2_, ZnS, and T321, as shown in Figure 18.

This study selected tungsten disulfide (WS_2_), zinc sulfide (ZnS), and isobutylene sulfide (T321) as modifying additives, aiming to systematically compare the effects and potential synergistic effects of different types of sulfur-containing compounds in improving the high-temperature tribological properties of gel-type greases. The specific reasons for selection are as follows:

1. Tungsten disulfide (WS_2_): As a typical layered transition metal disulfide, its interlayers are held together by weak van der Waals forces, making it easily shearable and thus providing excellent solid lubrication properties [26]. Compared to common MoS_2_, WS_2_ exhibits higher thermal stability (oxidation onset temperature > 400 °C), making it more suitable for the high-temperature operating conditions addressed in this study. We hypothesize that it can form a solid lubricating film with low shear strength on friction surfaces through physical adsorption and mechanical interlocking.
2. Zinc sulfide (ZnS): ZnS was selected for its status as an environmentally friendly solid additive. It is a wide-bandgap semiconductor material with stable chemical properties. During friction, ZnS may undergo tribochemical reactions to form protective films such as iron sulfide (FeS) on steel surfaces, thereby providing extreme pressure and anti-wear protection. Its mechanism of action may differ from that of conventional sulfurized olefin additives, providing a basis for comparison in this study.
3. Sulfurized Isobutylene (T321): This is a commercially widely used sulfur-phosphorus type extreme pressure anti-wear additive. It represents a class of mature liquid additives that function through friction chemical adsorption films. Selecting T321 allows for comparison with the two aforementioned solid additives (WS_2_, ZnS) and enables investigation into whether synergistic effects exist between solid lubrication and friction chemical lubrication.

By conducting single and blended studies on these three distinct sulfur-containing additives, this research aims to preliminarily identify the most effective additive combination for enhancing the high-temperature performance of lithium-based grease composites. This work also lays the experimental foundation for subsequent in-depth investigations into their synergistic mechanisms.

WS_2_ is industrially synthesized as powdered solid particles with a diameter of 1 μm (Shanghai Macklin Biochemical Co., Shanghai, China), as shown in Figure 18a. It forms a protective film on metal surfaces, reducing direct contact and effectively minimizing wear. WS_2_ exhibits exceptional stability at high temperatures, beginning decomposition at 450 °C and undergoing complete decomposition at 650 °C. Additionally, it maintains excellent lubricating properties in low-temperature environments. Relying on its outstanding adsorption capability, it forms a stable lubricating film on metal surfaces, providing effective protection even during cold starts. WS_2_’s high surface area and lubricity help smooth and repair surface imperfections, reducing heat generated by friction. Therefore, WS_2_ is a high-quality lubricant additive suitable for a wide temperature range.

ZnS is industrially synthesized as powdered solid particles with a diameter of 1 μm (Shanghai Aladdin Biochemical Technology Co., Shanghai, China), as shown in Figure 18b. It can form a protective metal salt layer in high-temperature environments, effectively preventing severe wear caused by direct contact between metal surfaces. ZnS also provides a degree of antioxidant protection by inhibiting free radical-induced oxidation reactions, thereby slowing down the aging process of oils. Additionally, ZnS particles can fill the minute pits on metal surfaces, acting to fill and smooth them. This further reduces the direct contact area between friction pairs, thereby lowering their wear rate.

The density of T321 is 1.2 g/cm^3^ (at 20 °C) (Lanzhou Lubricating Oil R&D Institute, Lanzhou, China), as shown in Figure 18c. It possesses outstanding anti-wear and anti-oxidation properties, forming a protective film on metal surfaces to reduce direct friction contact between metals, effectively minimizing wear and corrosion. T321 exhibits excellent thermal stability under high-temperature conditions, maintaining its lubricating properties in high-temperature environments to prevent premature oxidation or degradation of the lubricant. Additionally, T321 enhances the viscosity characteristics of lubricating oils, improving the durability and shear resistance of oil films, thereby increasing equipment operational efficiency and service life. T321 also exhibits strong low-temperature fluidity, ensuring the lubrication system operates normally in cold environments and effectively reducing wear during startup.

A review of the data showed that there is no chemical reaction between the three additives selected for incorporation into Mobil SHC 460WT lithium complex gel-like grease, so they can be safely mixed. In order to facilitate the subsequent research work, this study was conducted to optimize the formulation of Mobil SHC 460WT lithium complex gel-like grease. The optimized gel-like greases are numbered for ease of subsequent research and citation. This study selected a fixed additive concentration of 0.5 wt% for formulation optimization based on three primary considerations: First, the literature review indicates that adding 0.1% to 2.0% solid or liquid additives to gel-type greases to enhance their tribological properties represents a widely adopted and proven effective concentration range [25,26]. This concentration range ensures that the additive exerts a significant effect while avoiding potential issues such as colloidal structure instability, substantial cost increases, or the introduction of unnecessary side effects that may arise from excessive dosage. Secondly, the primary objective of this study is to systematically compare three distinct types of additives—WS_2_, ZnS, and T321—and to investigate the synergistic or antagonistic effects arising from their combined use. Setting a representative intermediate concentration (0.5%) provides a fair and comparable baseline for all samples, thereby enabling clearer identification of the mechanisms by which different additive combinations influence lubrication performance. Finally, based on preliminary exploration and industrial application experience, a 0.5% addition level is considered to strike a good balance between performance enhancement, formulation economics, and compatibility with base oils. This concentration is suitable for initial formulation screening studies targeting engineering applications. Subsequent studies may build upon this foundation to conduct more detailed concentration gradient optimization for optimal formulations (such as the WS_2_/T321 composite system). Table 4 shows the optimized ratio of Mobil SHC 460WT lithium complex gel-like grease.

To ensure uniform dispersion of additives and system stability, all modified grease samples were prepared according to the following unified procedure: First, the base grease was preheated to 60 ± 5 °C to reduce its consistency. Subsequently, precisely weighed additives (solid powders were pre-mixed with a small amount of base grease into a paste) were added. High-speed mechanical stirring (2000 ± 200 rpm) was applied at 60 ± 5 °C for 30 min. To achieve a more homogeneous colloidal structure and fully disperse the solid particles, the mixed grease was further refined through three passes on a three-roll mill (Changzhou Longxin Machinery Co., Changzhou, China). Finally, the samples were deaerated under vacuum at 60 °C for 15 min, followed by room-temperature standing for 24 h to restore structural stability. Upon completion, the samples were sealed and stored away from light.

### 4.2. Test Design

#### 4.2.1. Tribological Test Design

In the aforementioned testing machine, systematic tribological performance tests were conducted on eight different samples. The primary objective of the experiment is to conduct an in-depth analysis of the anti-friction and wear resistance properties of different samples by observing the trend of friction coefficient changes over time during the friction process. Simultaneously, after the test concludes, a detailed comparative analysis of the wear morphology and diameter of the test steel balls will be conducted to evaluate the protective effect on the steel ball surface and the wear suppression capability of different samples. The specific test equipment parameters and conditions are shown in Table 5. The design of these test parameters comprehensively references the principles of the ASTM Four-Ball Test Standard Method and incorporates engineering adjustments tailored to the high-temperature lubrication conditions of wind turbine main shaft bearings. To ensure data reliability and reproducibility, the friction and wear tests described above were repeated three times for each grease sample, and the average values along with standard deviations of both the friction coefficient and wear scar diameter are reported.

The selection of test parameters was designed to simulate the critical operating conditions of wind turbine spindle bearings, with the specific rationale as follows: The applied load (392 N), converted via the Hertzian contact stress in the four-ball test, corresponds to the medium-to-high stress level in the contact zone between the raceway and rolling elements under typical rated bearing load, serving to evaluate the extreme-pressure and anti-wear performance of the grease; the spindle speed (1200 rpm) represents the typical operating speed during medium-to-high power output, simulating a mixed lubrication regime with a certain slide-to-roll ratio; the test duration (3600 s) allows accelerated simulation of wear accumulation and grease performance evolution during the initial operating stage (equivalent to several hundred hours of actual service), thereby distinguishing the long-term stability of different formulations; and the temperature (80 °C) directly reflects the typical high-temperature condition inside the bearing under continuous full-load operation, used to assess the high-temperature lubrication effectiveness and thermo-oxidative stability of the grease.

#### 4.2.2. Rheological Test Design

Temperature is one of the key factors affecting the performance of gel-type grease in wind turbine main shaft bearings. Particularly under extreme temperature conditions, the rheological properties of gel-type grease undergo significant changes, directly impacting the lubrication effectiveness and operational stability of wind turbine bearings.

At extremely low temperatures, the fluidity of gel-like grease decreases significantly, and the strain value at the flow point increases, leading to an increase in the viscosity of the gel-like grease. This phenomenon may hinder the normal startup and operation of wind turbines under low-temperature conditions, potentially leading to issues such as accelerated bearing wear. Under extreme high temperatures, the fluidity of gel-type grease increases while its flow point strain value decreases. This may lead to premature breakdown of the gel structure, preventing the grease from effectively forming a stable lubricating film. Consequently, lubrication performance is reduced and bearing service life is shortened.

According to available data, the operating temperature range for wind turbine units is typically −20 to 80 °C, and the maximum operating temperature of bearings generally does not exceed 80 °C. Therefore, to comprehensively evaluate the performance of gel-like grease for wind turbine main shaft bearings under extreme temperatures, this study selected −20 °C and 80 °C as test temperature conditions. The focus was on testing key rheological parameters such as shear stress, shear rate, and viscosity–temperature behavior for different samples at varying temperatures. Through the acquisition and analysis of experimental data, this study aims to reveal the rheological behavior patterns of gel-type grease under extreme temperatures. This research provides a scientific basis for optimizing the formulation of gel-type grease used in wind turbine main shaft bearings, thereby further enhancing the operational stability and durability of these bearings.

To thoroughly investigate the rheological properties of different samples, this chapter systematically conducted flow characteristic and viscosity–temperature characteristic tests. In flow characteristic testing, by controlling the shear rate between 0.1 and 100 s^−1^, the variation in shear stress and viscosity with shear rate under sustained shear is analyzed for different samples. Based on this, viscosity–shear rate and shear stress–shear rate flow curves are plotted. Key rheological parameters include viscosity, shear stress, and shear rate.

**Figure 19:**
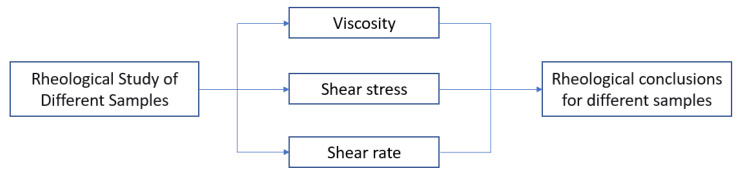
Experimental program for rheology of eight samples.

In the viscosity–temperature characteristic test, the shear rate was maintained constant at 50 s^−1^, while the temperature change rate was set to 1 °C/min to simulate temperature fluctuations under actual operating conditions. This yielded viscosity–temperature and shear stress–temperature flow curves for different samples under constant shear rate conditions. The primary rheological parameters were shear rate, viscosity, and temperature. All rheological tests were conducted in triplicate on the same fresh sample to ensure repeatability and accuracy of the data. The overall experimental workflow for the rheological characterization of the eight grease samples is summarized in Figure 19.

## Figures and Tables

**Table 1:** Shear stress values for eight samples at extreme temperatures.

Sample	−20 °C			80 °C		
	Initial Shear Stress/Pa	Ultimate Shear Stress/Pa	Percentage Change%	Initial Shear Stress/Pa	Ultimate Shear Stress/Pa	Percentage Change%
1	1184.3	24,362	19.57	113.74	336.37	1.96
2	676.67	15,553	21.98	116.59	301.93	1.59
3	436.56	20,001	44.82	114.79	302.13	1.63
4	732.51	20,005	26.31	98.38	307.01	2.12
5	898.02	15,521	16.28	114.89	307.32	1.67
6	288.17	6630.6	22.01	60.14	185.88	2.09
7	700.24	15,119	20.59	106.24	289.36	1.72
8	795.37	17,108	20.51	96.91	286.62	1.96

**Table 2:** Viscosity variation of eight samples.

Sample	−20~30 °C			30~80 °C		
	Initial Viscosity/Pa·s	Final Viscosity/Pa·s	Percentage Change/%	Initial Viscosity/Pa·s	Final Viscosity/Pa·s	Percentage Change/%
1	121.76	4.65	96.18	7.63	3.15	58.72
2	169.45	5.51	96.75	12.51	4.85	61.23
3	152.68	3.37	97.79	12.44	4.68	62.38
4	139.3	2.96	97.88	13.01	4.94	62.03
5	123.85	6.49	94.76	23.46	3.86	83.55
6	119.18	2.78	97.67	14.34	5.15	64.09
7	147.26	7.67	94.79	12.74	4.65	63.50
8	152.97	4.43	97.10	12.21	4.61	62.24

**Table 3:** Mobil SHC 460 WT Lithium Complex Grease Related Parameter.

Gel-like Grease Grades	NLGI Consistency Grades	Operating Temperature	Color (Visual)	Antioxidant Properties	Corrosion Resistance
Mobil SHC 460WT Lithium Complex Gel-Like Grease	1.5	−40~150 °C	red	high	high

**Table 4:** Optimized Ratio of Mobil SHC 460 WT Lithium Complex Grease.

Samples	Gel-like Grease	Food Additive	Ambient Condition	Additive Content (WT%)
1	Mobil SHC 460 WT Lithium Complex Gel-Like Grease	not have	Gelatinous	0.5
2		WS_2_		
3		ZnS		
4		T321		
5		WS_2_, ZnS		
6		WS_2_, T321		
7		ZnS, T321		
8		WS_2_, ZnS, T321		

**Table 5:** Test conditions.

Test Methods	Test Parameters
Equipment Model	MRS-10G Lever Type Four-Ball Friction Tester
Spindle speed r/min	1200
Temperature/°C	80
Load/N	392
Time/s	3600

## Data Availability

The original contributions presented in this study are included in the article. Further inquiries can be directed to the corresponding author.

## References

[1] Shangguan L., Xu Y. (2023). Design and Experimental Research on Centralized Lubrication and Waste Oil Recovery System for Wind Turbines. Appl. Sci..

[2] Peng H., Zhang H., Shangguan L., Fan Y. (2022). Review of Tribological Failure Analysis and Lubrication Technology Research of Wind Power Bearings. Polymers.

[3] Gbashi S.M., Olatunji O.O., Adedeji P.A., Madushele N. (2024). From academic to industrial research: A comparative review of advances in rolling element bearings for wind turbine main shaft. Eng. Fail. Anal..

[4] Han J. (2024). Additives for Lubricating Oil and Grease: Mechanism, Properties and Applications. Lubricants.

[5] Zhai G., Qin X., Yang X. (2021). Research on Real Working Condition Simulation and Performance Test of Wind Power Main Bearing Based on Test Bench. Math. Probl. Eng..

[6] Jiang Z., Huang X., Zhu H., Jiang R. (2022). A new method for contact characteristic analysis of the tapered roller bearing in wind turbine main shaft. Eng. Fail. Anal..

[7] Lin Y., Zhuang L., Xuan L., Hong W., Sun T., Yang P., Yan B. (2022). The Research on Condition Monitoring and Fault Diagnosis Method of Wind Power Spindle Bearing. J. Phys. Conf. Ser..

[8] Hart E., Stock A., Elderfield G., Elliott R., Brasseur J., Keller J., Guo Y., Song W. (2022). Impacts of wind field characteristics and non-steadydeterministic wind events on time-varying main-bearing loads. Wind Energy Sci..

[9] Zhao W., Zhang C., Wang J., Peyrano O., Gu F., Wang S. (2023). Research on main bearing life prediction of direct-drive wind turbine based on digital twin technology. Meas. Sci. Technol..

[10] Ren G., Sun X., Li W., Li H., Zhang L., Fan X., Li D., Zhu M. (2021). Improving the lubrication and anti-corrosion performance of polyurea grease via ingredient optimization. Friction.

[11] Negi R.S., Singh R.K., Datta S., Singh S.K. (2023). Investigation of Tribological Performance of Eco-friendly Pentaerythritol Tetraoleate Ester Based Calcium Complex Grease. Waste Biomass Valoriz..

[12] Kamel B.M., Awad M.N., Mobasher A., Hoziefa W. (2023). Lithium–Calcium Greases Having Carbon Nanotubes and Aluminum Oxide Base Nanoadditives: Preparation and Characteristics of Nanogrease. ACS Omega.

[13] Wang Y., Gao X., Lin J., Zhang P. (2022). Rheological and Frictional Properties of Lithium Complex Grease with Graphene Additives. Lubricants.

[14] Jiang Z., Huang X., Liu H., Zheng Z., Li S., Du S. (2022). Dynamic reliability analysis of main shaft bearings in wind turbines. Int. J. Mech. Sci..

[15] Zhao W., Jiang Z., Zhang P., Huang X. (2023). Reliability Sensitivity Analysis of Main Shaft Bearings of Wind Turbines Subject to Subsurface Stress. Machines.

[16] Jin S., Dong H., Chen J., Xie X., Guo M. (2022). Study on accelerated life tests for main shaft bearings in wind turbines. J. Mech. Sci. Technol..

[17] Mubashshir M., Shaukat A. (2019). The Role of Grease Composition and Rheology in Elastohydrodynamic Lubrication. Tribol. Lett..

[18] Zhou K., Che X., Wei C., Tang Z., Yu H., Wang D., Wang J., Zhang L. (2024). The Molecular Modeling, Simulation, and Design of Base Oils and Additives in Lubricating Oils: A Review. Processes.

[19] Wang X., Lv Z., Han Y., Wang J. (2024). Effect of Grease Composition on Impact-Sliding Wear. Lubricants.

[20] Li F., Liu D., Sun K., Hong S., Peng F., Zhang C., Tao T., Qin B. (2024). Stochastic and Extreme Scenario Generation of Wind Power and Supply– Demand Balance Analysis Considering Wind Power–Temperature Correlation. Electronics.

[21] Dhanola A., Garg H.C. (2020). Tribological challenges and advancements in wind turbine bearings: A review. Eng. Fail. Anal..

[22] Hu J., Hou J. (2025). An Energy-Efficient Fault Diagnosis Method for Subsea Main Shaft Bearings. J. Mar. Sci. Eng..

[23] Zheng M., Ren G., Wang S., Li Y., Xing M. (2024). Investigating the effect of overbased sulfonates on calcium sulfonate complex grease: Enhancements in physicochemical, rheological, and tribological properties. RSC Adv..

[24] Shamardi A., Soufi M.D., Ghobadian B., Almasi S. (2024). Synthesis of bio-based multi-purpose lithium grease from different vegetable oils: Drop point enhancement approach. Renew. Energy.

[25] Aboul-Enein A.A., Arafa E.I., Abdel-Azim S.M., Awadallah A.E. (2021). Synthesis of multiwalled carbon nanotubes from polyethylene waste to enhance the rheological behavior of lubricating grease. Fuller. Nanotub. Carbon Nanostruct..

[26] Zhao Z., Wang X., Hu Y., Li Z., Li L., Ye G. (2024). Investigation on the tribological properties and electrification performance of grease-lubricated triboelectric nanogenerators. Tribol. Int..

[27] Zhao J., Huang Y., He Y., Shi Y. (2021). Nanolubricant additives: A review. Friction.

[28] Grigoriev S., Soe T. (2022). The Influence of Surface Texturing of Ceramic and Superhard Cutting Tools on the Machining Process—A Review. Materials.

[29] Ding J., Feng A., Li X., Ding S., Liu L., Ren W. (2021). Properties, preparation, and application of tungsten disulfide: A review. J. Phys. D Appl. Phys..

[30] Wu C., Xiong R., Ni J., Yao L., Chen L., Li X. (2020). Effects of CuO nanoparticles on friction and vibration behaviors of grease on rolling bearing. Tribol. Int..

[31] Bond S., Jackson R.L., Mills G. (2024). The influence of various grease compositions and silver nanoparticle additives on electrically induced rolling-element bearing damage. Friction.

[32] Wu M., Han X., Tao Y., Pei Y. (2023). Lubrication reliability analysis of wind turbine main bearing in random wind field. Tribol. Int..

[33] Jiang Y., Liang B., Huang Z., Chen Z., Xu B. (2021). Prediction on Flow and Thermal Characteristics of Ultrathin Lubricant Film of Hydrodynamic Journal Bearing. Micromachines.

[34] Li K., Liu J., Chen W., Ye L., Zhang L. (2016). A Novel Bearing Lubricating Device Based on the Piezoelectric Micro-Jet. Appl. Sci..

[35] Jin X., Xia P., Liu Z., Ma W., Zhang P. (2022). Thermo-hybrid lubrication FSI-CFD model for the static characteristics of hybrid porous tilting pad bearings. Tribol. Int..

[36] Le D., Bui T. (2023). Analyzing the Effects of Lubrication Techniques on CNC Spindle Bearing Heat: An Experimental Investigation. Eng. Technol. Appl. Sci. Res..

[37] Meijer P., Lugt P. (2022). The Grease Worker and Its Applicability to Study Mechanical Aging of Lubricating Greases for Rolling BearingsP. Tribol. Trans..

[38] Lugt P., Berens F. (2022). The Grease Life Factor concept for ball bearings. Tribol. Int..

[39] Lugt P., Holgerson M., Reinholdsson P. (2023). Impact of oxidation on grease life in rolling bearings. Tribol. Int..

[40] Liu H., Dangl F., Lohner T., Stahl K. (2023). Numerical Visualization of Grease Flow in a Gearbox. Chin. J. Mech. Eng..

[41] Rosenkranz L., Richter S., Jacobs G., Mikitisin A., Mayer J., Stratmann A. (2021). Influence of temperature on wear performance of greases in rolling bearings. Ind. Lubr. Tribol..

[42] Hai Z.W., Qiang Y.X., Er S. (2018). Analysis of Dynamic Characteristics of Grease-Lubricated Tapered RollerBearings. Shock Vib..

[43] Wu P., He C., Chen G., Ren C. (2025). Determination of the equivalent friction coefficient of rolling bearings using the kinetic energy dissipation. Measurement.

[44] Zhang F., Chen M., Zhu Y., Zhang K., Li Q. (2023). A Review of Fault Diagnosis, Status Prediction, and Evaluation Technology for Wind Turbines. Energies.

[45] Kozdrach R., Radulski P. (2025). Application of chokeberry biochar as a modified additive to the vegetable lubricants: The tribological and rheological properties. Sci. Rep..

[46] Shen Y., Wang Y., Lin J., Zhang P., Gao X., Wang Z. (2023). Study on anti-wear and friction-reducing compounding additives in lithium greases. Ind. Lubr. Tribol..

[47] Olejarczyk K., Wikło M., Kołodziejczyk K. (2019). The cycloidal gearbox efficiency for different types of bearings—Sleeves vs. needle bearings. Proc. Inst. Mech. Eng. Part C J. Mech. Eng. Sci..

[48] Li C., Noman K., Liu Z., Feng K., Li Y. (2023). Optimal symbolic entropy: An adaptive feature extraction algorithm for condition monitoring of bearings. Inf. Fusion.

[49] Fan L., Wu Z., Yuan Y., Liu X., Sun W. (2025). Fatigue Life Prediction of Main Bearings in Wind Turbines Under Random Wind Speeds. Machines.

[50] de la Presilla R., Calderon-Salmeron G., Leckner J., Kitamura R., Sato K., Sasaki S., Glavatskih S. (2025). Lubricant design for oscillating rolling bearings: Greases, ionic liquids, and friction torque. Tribol. Int..

[51] Zhang S. (2025). Friction behavior of grease-lubricated rolling contact and its dependence on grease film under starvation. Tribol. Int..

[52] Jia J., Shi C., Yue P. (2025). Study on the interface and morphological control mechanism of calcium sulfonate grease thickener. Friction.

[53] Pan J., Wang J. (2025). An investigation of tribological properties of magnetorheological grease with thermomagnetic coupling. J. Magn. Magn. Mater..

[54] Ding P., Cao W. (2025). The influence of mechanical shearing on the thickener microstructure and properties of Polyurea greases. Tribol. Int..

